# Elastic Tape Improved Shoulder Joint Position Sense in Chronic Hemiparetic Subjects: A Randomized Sham-Controlled Crossover Study

**DOI:** 10.1371/journal.pone.0170368

**Published:** 2017-01-18

**Authors:** Gabriela Lopes dos Santos, Matheus Bragança Souza, Kaat Desloovere, Thiago Luiz Russo

**Affiliations:** 1 Laboratory of Neurological Physiotherapy Research, Department of Physical Therapy, Federal University of São Carlos (UFSCar), São Carlos, Brazil; 2 Department of Rehabilitation Sciences, Faculty of Kinesiology and Rehabilitation Sciences, Katholieke Universiteit Leuven, Leuven, Belgium; 3 Clinical Motion Analysis Laboratory (CERM), University Hospital Pellenberg, Pellenberg, Belgium; Cardiff University, UNITED KINGDOM

## Abstract

**Background:**

Elastic tape has been widely used in clinical practice in order to improve upper limb (UL) sensibility. However, there is little evidence that supports this type of intervention in stroke patients.

**Objective:**

To verify the effect of elastic tape, applied to the paretic shoulder, on joint position sense (JPS) during abduction and flexion in subjects with chronic hemiparesis compared to sham tape (non-elastic tape). Furthermore, to verify if this potential effect is correlated to shoulder subluxation measurements and sensorimotor impairment.

**Methods:**

A crossover and sham-controlled study was conducted with post-stroke patients who were randomly allocated into two groups: 1) those who received Sham Tape (ST) first and after one month they received Elastic Tape (ET); 2) those who received Elastic Tape (ET) first and after one month they received Sham Tape (ST). The JPS was evaluated using a dynamometer. The absolute error for shoulder abduction and flexion at 30° and 60° was calculated. Sensorimotor impairment was determined by Fugl-Meyer, and shoulder subluxation was measured using a caliper.

**Results:**

Thirteen hemiparetic subjects (average time since stroke 75.23 months) participated in the study. At baseline (before interventions), the groups were not different for abduction at 30° (p = 0.805; p = 0.951), and 60° (p = 0.509; p = 0.799), or flexion at 30° (p = 0.872; p = 0.897) and 60° (p = 0.853; p = 0.970). For the ET group, differences between pre and post-elastic tape for abduction at 30° (p<0.010) and 60° (p<0.010), and flexion at 30° p<0.010) and 60° (p<0.010) were observed. For the ST group, differences were also observed between pre and post-elastic tape for abduction at 30° (p<0.010) and 60° (p<0.010), and flexion at 30° (p<0.010,) and 60° (p<0.010). Potential effects were only correlated with shoulder subluxation during abduction at 30° (p = 0.001, r = -0.92) and 60° (p = 0.020, r = -0.75).

**Conclusion:**

Elastic tape improved shoulder JPS of subjects with chronic hemiparesis regardless of the level of UL sensorimotor impairment. However, this improvement was influenced by the subluxation degree at abduction.

## Introduction

Stroke is one of the leading causes of death and disability in adults [1, 2]. Approximately 70% of post-stroke patients have sensorimotor deficits in the upper limb (UL), which result in contralateral hemiparesis injury. [3]. These sensorimotor deficits can include somatosensory alterations, which impair movement control and joint stability [4, 5]. An important subsystem of the somatosensory system involves proprioception [6], which consists of afferent information originating from mechanoreceptors [6–8]. Proprioception can be divided into three submodalities, i.e. kinesthesia, sense of tension or force and joint position sense (JPS) [4–6, 8, 9]. Proprioceptive deficits impair feedback and feedforward control, which negatively influence joint stability, acuity and coordination movements [4, 6], mainly small or precise movements [10], as well as motor skill acquisition [8, 10].

Fifty percent of post-stroke subjects present proprioceptive deficits in the UL [11]. According to previous studies, subjects with chronic hemiparesis presented bilateral deficits of kinesthesia during internal and external shoulder rotation [12, 13], as well as bilateral deficits of JPS during movement abduction and flexion of the shoulder. These deficits are related to the degree of shoulder subluxation [14]. Moreover, these proprioceptive deficits are also associated with UL motor recovery and function [15, 16], which impair the performance of activities of daily living [10], and possibly restrict participation and quality of life. Therefore, treating proprioceptive impairments is one of the main objectives in rehabilitation programs for stroke patients [17, 18].

Given these clinical findings, some strategies to improve proprioception have been used in clinical practice, such as augmentation of somatosensory information via passive techniques that involves manual therapy, soft tissue techniques and taping or brace applications [4, 19]. Taping consists of an adjunct technique, which uses an elastic adhesive tape over the skin in order to stimulate mechanoreceptors via continuous skin stretching and compression during joint motion [4, 19]. Based on accepted principles in neuroscience, it can be hypothesized that these afferent stimuli are transmitted to the contralateral area of the somatosensory cortex, which integrates information from different sensory and motor modalities [20]. One taping technique widely used in clinical practice is the Kinesio Taping method [21].

According to previous studies, taping increased electromyographic activity and improved the shoulder joint position sense of healthy individuals [22, 23]. Moreover, taping helped to perform simple and proprioceptive activities during knee extension in healthy subjects, which were associated with more bilateral activation in the primary sensorimotor cortex and primary sensory cortex, and less bilateral activation in the cingulate motor area and cerebellum [24]. Thus, these results demonstrated that taping can influence neural activation, as well as provide biomechanical support, i.e., improving shoulder girdle stability [23, 24]. Regarding stroke patients, a systematic review [25] highlighted that the effects of taping on pain intensity, muscle tone, range of motion and strength were inconclusive, and that there was insufficient evidence related to activity and participation. Hence, the authors concluded that there is a need for more in-depth research that can verify the taping effects on this population.

Although systematic review [25] and studies have shown that elastic tape does not reduce shoulder pain [26, 27] and subluxation [28], nor does it increase the range of motion [26, 27], motor function and functionality in post-stroke subjects [26, 27], other studies observed opposite effects of the UL from the same population, such as reduced pain, improvements in range of motion [28, 29], motor function and functionality [28, 30] after intervening with elastic tape. Thus, the literature supported the lack of consensus of the effects of elastic tape used in the UL of post-stroke subjects, requiring more studies. Furthermore, to the best of our knowledge, there is no evidence regarding the effect of taping on proprioception (joint position sense) in this population. Our study will test if taping is able to provide any improvement of the sensorial feedback in the shoulders of chronic post-stroke subjects.

The main purpose of this study was to verify the effect of the elastic tape, used on the paretic shoulder (anterior, middle, and posterior deltoid), on the JPS of the paretic side during abduction and flexion in chronic hemiparetic subjects, compared to rigid tape (sham). Secondly, another aim was to verify if the possible improvement of shoulder girdle stability provided by the elastic tape on the paretic side could influence the JPS of the non-paretic side. Thirdly, another objective was to verify if this potential effect (difference between pre and post intervention after elastic tape) on the paretic shoulder was correlated to the baseline shoulder subluxation measurements and sensorimotor impairment. Therefore, it can be observed whether there is a relationship between the amount of deficits and response to the treatment. The following hypotheses were tested: (1) elastic tape improves JPS on the paretic side, (2) elastic tape improves JPS on the non-paretic side by increasing the proximal stability and (3) this change is negatively correlated to the baseline subluxation grade and sensorimotor impairment.

## Materials and Methods

### Experimental design

This study presents a randomized sham-controlled crossover study, which was conducted with chronic hemiparetic subjects at a center (UFSCar, Brazil). Patients were recruited from lists acquired from the rehabilitation center and the University Hospital of São Carlos, São Paulo, Brazil. Patients were not involved in any rehabilitation program. The research activities of this study received ethical approval by the Ethics Committee in Brazil (Number: 966636) and was registered in the Clinical Trials (URL: http://www.clinicaltrials.gov. Unique identifier: NCT02390115). Written informed consent was obtained from each patient prior to taking part in the study. All data regarding the trial for this intervention were registered. However, the registration date was retrospective to the participants′ enrollment due to insufficient information for registration, for example, little information regarding the tape application protocol. The CONSORT checklist, study protocol and individual data are available as supporting information (S1, S2, and S3 Files, respectively).

Assessments were divided into three days and all evaluations were carried out at the Federal University of São Carlos (UFSCar), Brazil. On the first day, screening to select the sample and clinical assessment was done. In the first and second sessions, the JPS was assessed, followed by a wash-out period of one month between sessions. In both sessions, the JPS test was run without and with intervention (elastic or sham tape). Patients were randomly assigned to one of two groups using sealed opaque envelopes to receive the Sham Tape (ST) first or the Elastic Tape (ET) first. An independent staff member prepared the envelopes. However, the assessor and patient were not blinded when the intervention took place due to the color of the tape and not being able to cover the limb, which could generate more sensory input and impair the test. Thus, before the test, the assessor read a standard text to the patient: “I will put the tape on your shoulder and you will do a test, which I will explain later”. A schematic representation of the experimental design is shown in S1 Fig.

### Participants

Considering the hemiparetic subjects, the following inclusion criteria were used: (1) unilateral ischemic stroke of either hemisphere with lesions restricted to the anterior vascular territory (anterior and medium cerebral arteries) observed in the medical report of the MRI; (2) at least 6 months post stroke; (3) spasticity for shoulder abductor and flexor muscle level of less than 3 on the Modified Ashworth Scale (MAS); (4) mild or moderate UL sensorimotor impairment (score of ≥ 30 on the Fugl-Meyer UL motor part) [31]; (5) proper trunk control, defined as the ability to remain in a seated position without support for the trunk and/or of the arms for one minute, and a minimum score on the Mini-Mental State Examination, according to the subject’s educational level [32]. Individuals who had more than one stroke could be included if the vascular accident involved the same hemisphere.

The following exclusion criteria were applied: diabetes mellitus, ulcers or skin lesions; elastic tape adverse reactions (redness and itching); serious cardiovascular or peripheral vascular disease (heart failure, arrhythmias, angina pectoris or myocardial infarction); other orthopedic or neurological diseases that affected the data collection were; cognitive or communication impairments; shoulder pain during the test; history of muscle or joint injuries at the shoulder complex or cervical joints (fractures or surgery); abnormal sensitivity, understanding of aphasia, apraxia, hemineglect and/ or plegia. In addition, individuals with other neurologic diseases, hemorrhagic stroke or any injury to the occipital lobe, brainstem or cerebellum were also excluded. Furthermore, individuals with a passive range of motion of the shoulder lower than 90° flexion, 30° extension and adduction were excluded. These ranges of motion were necessary to standardize the application of elastic tape.

### Clinical assessment

One evaluator performed the clinical assessment. Participants were submitted to an interview that included collecting personal data, a physical examination (anthropometric data), and investigating the upper extremity sensorimotor impairment adopting the Fugl-Meyer Assessment (FMA) [33]. Furthermore, the scores for motor function, sensitivity and coordination/velocity of the FMA were calculated. The presence of shoulder subluxation was quantified using a caliper. Based on the distance between the lateral edge of the acromion and the upper edge of the humeral head, the subluxation was graded as 0, 1+, 2+ or 3+ for distances of <0.5 cm, 0.5 to 1 cm, 1 to 2 cm, or >2 cm, respectively [14, 34]. The same assessor took this measurement on two different days in exactly the same way (clinical and first proprioception assessment) in order to perform the intra-rater reliability measurement. The reliability for the subluxation measurement using a caliper was the Intraclass Correlation Coefficient, ICC (2, 1) = 0.97; 95% Confidence Interval [0.50–0.99]; Standard Error of Measurement (SEM) = 0.10 cm. The side of the lesion was verified in the MRI medical report [35, 36]. Manual preference was assessed by the Edinburgh Handedness Inventory [37], considering the preference before the stroke.

### Joint Position Sense (JPS) assessment

The JPS assessment was carried out using a dynamometer (Biodex Multi-joint System 3, Biodex Medical System Inc., New York). Before each test, the dynamometer was calibrated according to the manufacturer′s guidelines. The subjects were positioned in the dynamometer seat with 90° of hip flexion, and the pelvis and trunk were stabilized using straps. The attachment was fixed at the distal part of the arm [14].

The following instructions were given to the patient: (1) the dynamometer will move your arm to a specific position, (2) you will remain in this position for ten seconds, observe where you arm is positioned, (3) the dynamometer will return your arm to the starting position, (4) the dynamometer will move your arm again, and (5) press the button to stop the machine when you notice that your arm has reached the previous position [14]. The stop button was held in the non-paretic hand. Initially, one familiarization trial was conducted. During the test, participants were blindfolded to rule out visual cues and no communication was allowed [38, 39]. The dynamometer moved each subject’s upper extremity passively at a fixed rate of 2.0° per second from the starting position (0° of abduction or flexion) to the reference positions (30° and 60° of abduction and then 30° and 60° of flexion). The absolute error (in degrees) was calculated as the difference between the indicated and reference positions [40].

The test was carried out three times for each limb (paretic or non-paretic), movement (abduction or flexion) and angle (30° or 60°), and then after, as well as before elastic or sham tape intervention. Twenty-four movements were performed for each limb, and the absolute error was the average of three attempts. The order of movements and angles was randomized to prevent possible learning effects, however the assessments always started with the paretic limb.

### Intervention

A physiotherapist who was certified in Kinesio Taping placed the tapes (sham and elastic) on the paretic shoulder. Blue Kinesio^®^ Tex Gold Finger Print tape (5 cm wide) was used for the elastic tape intervention and Cremer tape strips (5 cm wide) (Cremer S/A, São Paulo, Brazil) were used for the sham intervention. After putting on the tapes, and before re-evaluating the JPS, the patients remained seated for 10 minutes. Then, the JPS test was performed again with the intervention (sham or elastic tape). A previous study showed a short-time effect after 10 minutes using the elastic tape [41].

To attach the elastic tape, the acromioclavicular joint was considered as the initial anchor and one point immediately below the insertion of the deltoid muscle as the final anchor. The first tape was placed on the anterior portion of the deltoid with the shoulder at 30° passive extension. The second tape was placed on the middle portion of the deltoid with the shoulder at 30° passive horizontal adduction. To place the third tape on the posterior deltoid, the limb was positioned at 90° of passive flexion of the shoulder (data in S2 Fig). The elastic tape tension was described as “paper tension” and was equivalent to 10–15% of the total elastic tape tension, i.e., no tension was applied to the tape by the therapist [42]. According to the Kinesio Taping method, the tape application from origin to insertion with 10–15% of tension can facilitate muscle function and provide more support by increasing the sensory stimulation without performing the functional correction (mechanical support) [43]. Furthermore, also according to the method, applying the tape with the stretched muscle generates convolutions on the skin when the patient returns to neutral position, which can increase the sensory input [44]. The sham tape was placed similarly to the elastic tape with the patient in the same position.

### Perceived effects

An assessment of the perceived effects was carried out during the first session after the JPS for both groups. The aim of this assessment was to verify whether the applied sham intervention was a plausible comparator for this study. Three questions were asked to the volunteers, which were the following: "Do you think the effects of the treatment that you received: 1—improved your perception of the limb in space? 2—improved using the limb? 3- improved the sensitivity of the limb?", with response options of yes or no. Each response was ranked as 0 or 1, corresponding to no or yes, respectively, and resulting in a total score ranging from 0 (no treatment effect) to 3 (maximum treatment effect) [45, 46].

### Outcome measures

The primary outcome variable in this study was shoulder JPS impairment, expressed by the absolute error in degrees for paretic and non-paretic limbs measured before and after interventions in the first and second sessions. Secondary outcome variables were the grades of subluxation measured using a caliper, the upper extremity sensorimotor impairment quantified by the Fugl-Meyer Assessment (FMA) and the scores for motor function, sensitivity and coordination/velocity measured by the FMA subscales. These secondary outcome variables were measured on the first day during clinical assessment. Another secondary variable was the subjective perception of the effects measured by the number of patients who received a different total score (0, 1, 2 or 3) after the JPS test during the first session.

### Statistical analysis

A mixed model, two-way analysis of variance (group and evaluation time) with repeated measurements (evaluation time: pre- and post-sham or elastic tape) with Bonferroni’s correction was used to examine the effect of group-by-evaluation time interaction, group (sham tape first and elastic tape first), and evaluation time (after and before sham and elastic tape). This analysis was performed for the paretic and non-paretic sides for both groups. Furthermore, partial eta (*η*^2^) was used to determine the effect size of the interaction and quantify the proportion of total variance (from 0 to 1) which explains the dependent variable [47, 48]. By convention, an *η*^2^ around 0.2, 0.5, and 0.8 was considered small, medium, and large, respectively [49]. The effect of elastic tape in each group was estimated as the difference of means pre and post intervention (effect size: ES) and 95% confidence interval (95% CI) [50].

The difference between the absolute error average at pre and post-elastic tape intervention was calculated for the shoulder abduction and flexion, and was referred to as the ‘potential effect’. This change in each angle (30° and 60°) per movement (abduction and flexion) was correlated to the subluxation grade, total FMA score, subscale scores for the motor function, sensitivity, and coordination/velocity of the FMA using the Spearman test. The magnitude of correlations was analyzed based on the Munro classification [51]: low (0.26–0.49), moderate (0.50–0.69), high (0.70–0.89), and very high (0.90–1.00). All statistical tests were carried out using SPSS software version 17.0 (SPSS Inc, Chicago, IL, USA), and a significance level was set at 0.05.

## Results

### Participants

The sample size was calculated using pilot data from four subjects with chronic hemiparesis from the elastic tape group and four subjects with chronic hemiparesis from the sham tape group using G.Power 3.1 software [52]. For this calculation, the absolute error was considered during abduction at 30° because it was the variable that presented the highest sample size after calculating it. The average and standard deviation from these pilot data were presented in S1 Table. For this calculation, the F-test (repeated measures ANOVA, within and between factors) was used and a power of 0.80 and alpha of 0.05 were considered. In addition, a loss of 15% of the data were considered, requiring a total sample size of 10.

**Fig 1:**
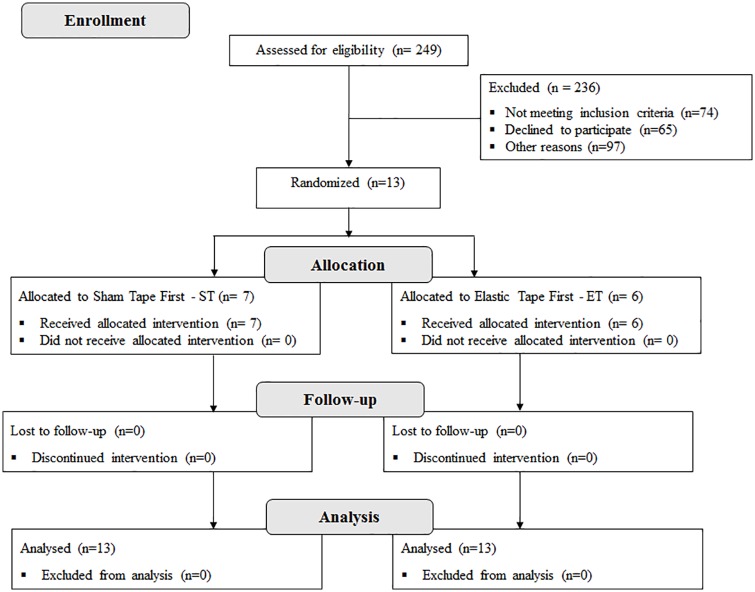
Flow diagram of the study.

From July 2014 to July 2015, 249 subjects with chronic hemiparesis from a hospital list in São Carlos were assessed for eligibility. However, 236 participants were excluded. Among the excluded patients, 65 declined to participate, 74 did not meet the inclusion criteria and 97 were excluded for other reasons (they did not answer the phone or the number did not exist). Thus, 13 subjects (3 women and 10 men) were randomly allocated to the two groups: sham followed by elastic tape (n = 7, ST) or elastic tape followed by sham (n = 6, ET). All included patients completed the crossover experiment. The data analysis was successfully conducted for all the participants (Fig 1).

The demographic characteristics of the participants are presented in Table 1. The range of age was 45 to 73 years with a BMI within the normal range. The range of post-stroke time was 24 to 158 months. All patients were right-handed before the stroke. However, the stroke occurred approximately at the same proportion in the right and left hemispheres. Shoulder assessment revealed that eleven patients presented subluxation with muscle tone of 0, 1 or 1+.

**Table 1:** Demographic characteristics of participants.

Demographics outcomes	Values
Age (years)	59.46 (±8.88)
Weight (Kg)	67.43 (±12.68)
Height (m)	1.66 (±0.10)
BMI (Kg/m^2^)	24.37 (±2.64)
Time post-stroke (months, min-max)	75.23 (24–158)
Dominant side (R/ L)	13/0
Hemiparesis side (R/L)	6/7
Shoulder subluxation grade (0/1+/2+/3+)	4/2/6/1
Passive ROM of shoulder abduction (°)	132.31 (±24.05)
Passive ROM of shoulder flexion (°)	122.69 (±31.69)
MAS of shoulder abduction (0/1/1+/2/3/4)	6/4/3/0/0/0
MAS of shoulder flexion (0/1/1+/2/3/4)	6/4/3/0/0/0
Total score of FMA (median, min-max)	49 (32–57)
Subscale score of motor function (median, min-max)	43 (28–51)
Subscale score of sensibility (median, min-max)	8 (3–12)
Subscale score of coordination/velocity (median, min-max)	5 (3–6)

BMI: Body Mass Index. R: Right. L: Left. MAS: Modified Ashworth Scale. FMA: Fugl-Meyer Assessment. Data expressed as mean and standard deviation, except time post-stroke expressed as mean (minimum-maximum), total and subscales score of FMA as median (maximum-minimum).

### Effects of elastic tape on paretic side

**Fig 2:**
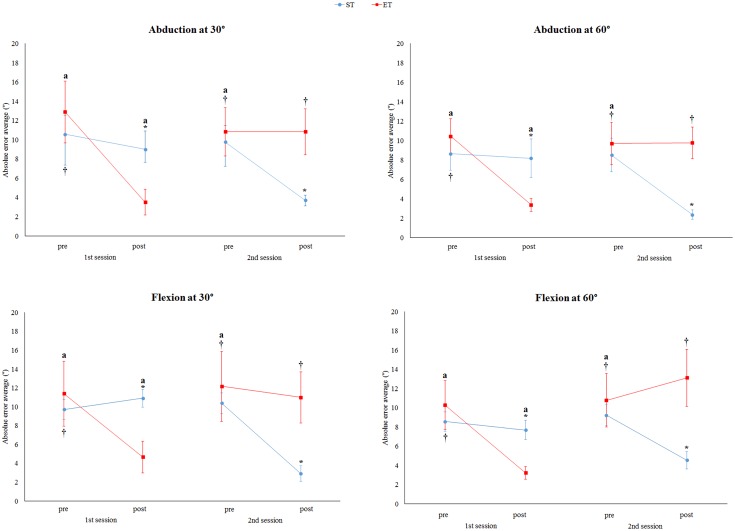
Average absolute error of paretic side for abduction and flexion at 30° and 60° for sham tape first (ST) and elastic tape first (ET) pre and post-intervention for the patient group. No differences at baseline (pre-intervention in the first and second sessions) between the ST and ET were observed for all movements and angles. For ST, in the post-intervention in the second session (post-elastic tape), a lower absolute error was observed compared to another evaluation time. For ET, in the post-intervention in the first session (post-elastic tape), a lower absolute error was observed compared to another evaluation time. *Significant differences compared to ET (p<0.05). †For the ET group, significant differences compared to the post-intervention in the first session (p<0.05). ªFor the ST group, significant differences compared to the post-intervention in the second session (p<0.05). Data were expressed as the mean and standard error.

The results revealed interaction between the group (sham first and elastic tape first) and evaluation time (pre and post-intervention) for abduction at 30° (F = 57.21, p<0.001, *η*^2^ = 0.51), abduction at 60° (F = 89.35, p<0.001, *η*^2^ = 0.58), flexion at 30° (F = 45.07, p<0.001, *η*^2^ = 0.59), and flexion at 60° (F = 41.09, p<0.001, *η*^2^ = 0.55) (Fig 2).

For both groups, there was an elastic tape effect characterized by a decrease in the average absolute error for all movements and angles (Fig 2). For the ET group, the data analysis highlighted differences between pre and post-elastic tape in the first session for abduction at 30° (ES: 9.39, 95% CI: 4.90–13.87, p<0.001), abduction at 60° (ES: 7.05, 95% CI: 4.34–9.76, p<0.001), flexion at 30° (ES: 6.72, 95% CI: 2.24–11.19, p<0.001), and flexion at 60° (ES: 7.06, 95% CI: 2.53–11.59, p<0.001,). For the ST group, differences between pre and post-elastic tape in the second session for abduction at 30° (ES: 6.850, 95% CI: 2.73–9.35, p<0.001), abduction at 60° (ES: 5.14, 95% CI: 1.60–8.69, p<0.001), flexion at 30° (ES: 6.47, 95% CI: 3.01–9.93, p<0.001), and flexion at 60° (ES: 4.70, 95% CI: 3.18–6.21, p<0.001) were found.

In contrast, there was no effect of sham tape intervention for both groups during all the movements and angles (Fig 2). For the ET group, no differences between pre and post-sham tape in the second session for abduction at 30° (p = 1.00), abduction at 60° (p = 1.00), flexion at 30° (p = 1.00) and flexion at 60° (p = 0.398) were observed. These differences were also not observed for the ST group in the first session during abduction at 30° (p = 0.554), abduction at 60° (p = 0.408), flexion at 30° (p = 1.00), and flexion at 60° (p = 1.00).

In addition, no differences between the pre-intervention of both sessions for all groups were observed (Fig 2), demonstrating that the order of intervention did not influence the results. For the ET group, no differences between the pre-intervention in the first and second sessions were observed for abduction at 30° (p = 0.249), abduction at 60° (p = 0.263), flexion at 30° (p = 0.425) and flexion at 60° (p = 1.00). For the ST group, no differences between pre-intervention in the first and second sessions were observed for abduction at 30° (p = 1.00), abduction at 60° (p = 1.00), flexion at 30° (p = 0.194) and flexion at 60° (p = 0.639).

After comparing the groups at baseline in both sessions, no differences were observed between the ET and ST groups for all the movements and angles (Fig 2). In the first session before intervention, the ST and ET were not different for abduction at 30° (p = 0.805), abduction at 60° (p = 0.509), flexion at 30° (p = 0.872) and flexion at 60° (p = 0.853). In the second session before intervention, the ST and ET were not different for abduction at 30° (p = 0.951), abduction at 60° (p = 0.799), flexion at 30° (p = 0.897) and flexion at 60° (p = 0.970). However, the groups were different after intervention in the first and second sessions for abduction at 30° (p = 0.022; p = 0.010), abduction at 60° (p = 0.020; p = 0.001), flexion at 30° (p = 0.004; p = 0.018) and flexion at 60° (p = 0.011; p = 0.014).

### Correlations between effects of elastic tape for paretic side

The correlations between the potential effect (difference between average absolute error at pre and post elastic tape) during flexion at 30° with subluxation measurement (p = 0.339), the total score of FMA (p = 0.409), the subscale score of motor function (p = 0.502), the sensibility (p = 0.720), and the coordination/velocity (p = 0.502) did not reach statistical significance for this sample size. Moreover, the correlation between the potential effect during flexion at 60° with subluxation measurement (p = 0.779), the total score of FMA (p = 0.137), the subscale score of motor function (p = 0.118), the sensibility (p = 0.671), and the coordination/velocity (p = 0.118) did not reach statistical significance.

**Fig 3:**
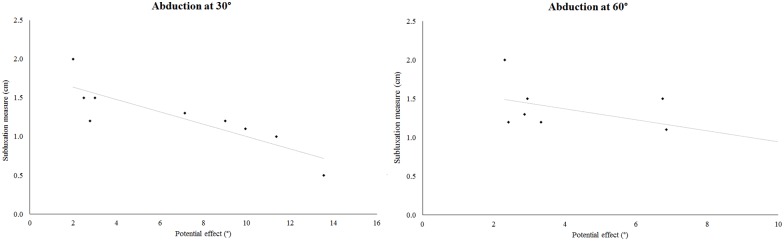
Correlations of the potential effect during abduction at 30° and 60° with the shoulder subluxation grade. A significant high correlation was observed during abduction at 30°, while a (non-significant) moderate correlation was found at 60°.

For abduction at 30°, no correlations were found with the total score of FMA (p = 0.470), the subscale score of motor function (p = 0.423), the sensibility (p = 0.645) and the coordination/velocity (p = 0.423). The correlation between the potential effect during abduction at 60° with the total score of FMA (p = 0.481), the subscale score of motor function (p = 0.401), the sensibility (p = 0.811) and the coordination/velocity (p = 0.401) was not observed. However, there was a significant negative and high correlation between the baseline subluxation measurement with the potential effect during abduction at 30° (p = 0.001, r = -0.92; Fig 3) and abduction at 60° (p = 0.020, r = -0.75; Fig 3).

### Effects of elastic tape for non-paretic side

**Fig 4:**
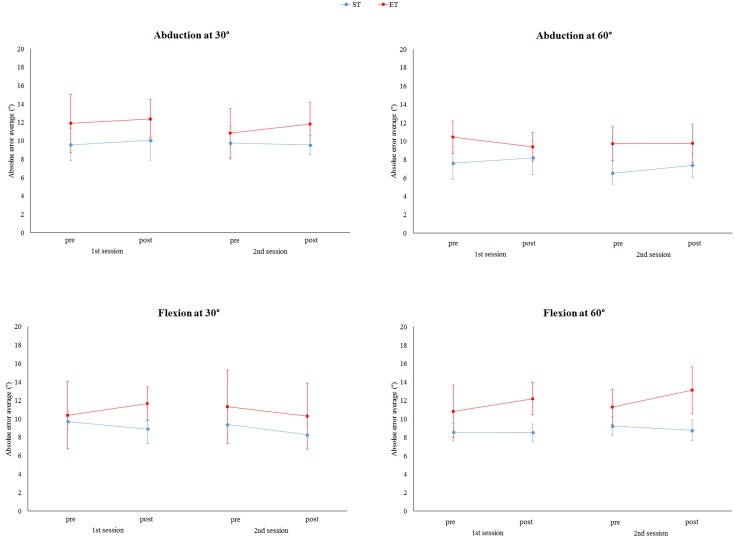
Average absolute error of non-paretic side for abduction and flexion at 30° and 60° for sham tape first (ST) and elastic tape first (ET) pre and post-intervention. No differences were found between the ST and ET in pre and post-interventions in the second and first sessions for all movements and angles (p>0.05). In addition, for both groups, no differences between the time evaluation were observed for all the movements and angles. Data were expressed as mean and standard errors.

Both interventions (elastic and sham tape) did not present effects on the non-paretic side for both groups (Fig 4). No interactions between the group (sham first and elastic tape first) and evaluation time (pre and post-intervention) for abduction at 30° (F = 1.19, p = 0.322), abduction at 60° (F = 2.38, p = 0.087), flexion at 30° (F = 3.06, p = 0.086) and flexion at 60° (F = 1.69, p = 0.214) were observed. In addition, no differences between the evaluation time for abduction at 30° (F = 1.53, p = 0.239), abduction at 60° (F = 1.87, p = 0.154), flexion at 30° (F = 3.06, p = 0.086) and flexion at 60° (F = 1.40, p = 0.268) were found. Furthermore, no differences between the ET and ST groups were observed for abduction at 30° (F = 0.31, p = 0.587), abduction at 60° (F = 1.07, p = 0.158), flexion at 30° (F = 0.00, p = 0.986) and flexion at 60° (F = 1.86, p = 0.200)

### Perceived effects

**Fig 5:**
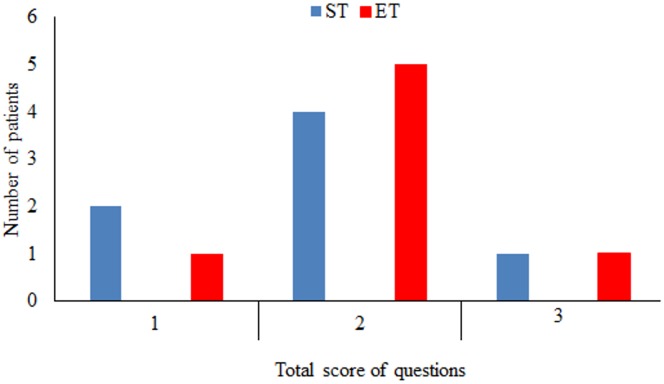
Number of patients with total score of 1, 2, and 3 in questions about perceived effects after the JPS test for sham tape first (ST) and elastic tape first (ET) during the first session. The number of patients in each total score was similar after the interventions for both groups, demonstrating the analogous subject’s feelings, regardless of the intervention.

Fig 5 shows that the number of patients in each total score was similar after intervention for both groups, demonstrating that the sham intervention was a plausible comparator for this study.

### Adverse effects

No adverse effects (redness or itching) were observed during data collection.

## Discussion

Although elastic tape has been widely used as a therapeutic tool in clinical practice, there is little evidence that supports this type of intervention in stroke patients. Furthermore, in accordance with systematic reviews and meta-analyses, the current available evidence is of low quality and insufficient to draw conclusions about the effects of elastic tape on different populations and/or pathologies [25, 53–57]. Thus, to the best of our knowledge, this is the first randomized sham-controlled crossover study that has verified immediate effects of elastic tape applied to the paretic shoulder, on JPS of the paretic and non-paretic side during abduction and flexion in subjects with chronic hemiparesis, compared to rigid tape.

The results of the present study revealed that elastic tape only improves JPS on the paretic side for all analyzed movements and angles characterized by a decreased absolute error. These findings confirm the first hypothesis. However, these results are not in line with previous studies that did not observe any effects of elastic tape on shoulder proprioception in athletes [58, 59] and healthy subjects [60]. These conflicting results may be partially explained by differences between the evaluated populations and assessment tools, such as the use of the inclinometer versus an optoelectronic system for three-dimensional analyses. On the other hand, our results are in agreement with previous studies that used the same measurements on the knees of healthy subjects. These studies observed an improvement in JPS after using elastic tape in participants with poor proprioception compared to the good proprioception group [61, 62]. In addition, facilitators′ effects were also observed when elastic tape was used on the knees of healthy subjects [24] and in patients with patellofemoral pain syndrome [63], and on the shoulders of healthy subjects [22, 23].

Based on previous literature [24, 61–63] and neuroscience knowledge [20], it can be suggested that a possible explanation for the effect of elastic taping may be related to the neural activation and biomechanics support. Elastic tape produces tactile stimulation, which increases sensory input from mechanoreceptors to the cortex contralateral primary somatosensory via thalamus [4, 19, 20]. The primary somatosensory cortex has a connection with multimodal association areas, which integrates information from different sensory modalities such as visual and proprioceptive information. These areas are linked to multimodal motor association areas that transform sensory information into planned movements and calculate the necessary programs for movements (feedforward and feedback control). In addition, these motor multimodal areas are linked to the primary motor cortex and premotor areas [20]. This possible central neural influence of the elastic tape was demonstrated by a previous study [24], that observed the increase in bilateral activation of sensory and sensorimotor areas and bilateral decrease in areas related to decision making and planning, and coordination of the some aspects of proprioception, such as cerebellum and the cingulate motor area.

Because the sham intervention (rigid tape) had no effect in the position sense, it can be suggested that the effects of the elastic tape may be due to its elastic property. This property can produce mechanical changes on the skin, such as stretching and compression, thereby increasing the sensory input [4, 19, 24]. It is worth highlighting that the sham intervention was considered a plausible comparator for this study. Finally, as there were no changes in the JPS between the two evaluations on the non-paretic (i.e. non-treated) side, it can be concluded that there was no learning effect. Moreover, although the elastic tape may improve shoulder girdle stability [23], which can improve body position and perception bilaterally, these results demonstrate that the immediate effects of the elastic tape were limited to the applied part of the body. Furthermore, while previous study demonstrated that elastic tape provided a bilateral activation in the sensorimotor cortex [24], it did not reflect immediate changes on the shoulder JPS on the non-paretic side, reinforcing that short-term effects of the tape are local.

Another important result of this study is related to a negative correlation between the potential effect (difference between the absolute average error in the pre and post elastic tape intervention) and the measurement of shoulder subluxation during abduction. According to the literature, subluxation impairs the abduction motion more than the flexion motion, due to anatomical and biomechanical aspects [64, 65]. However, no correlations between the sensorimotor impairment (FMA score) for all the movements and angles were observed, which refutes the second hypothesis of the study. The lack of correlation demonstrated that patients with mild or moderate UL sensorimotor impairment can benefit from using elastic tape, regardless of the impairment level.

Overall, the results of this study demonstrated that elastic tape could be considered as an important intervention strategy for post-stroke subjects in chronic phases, regardless of the UL sensorimotor impairment level. Apart from the relationship between the effect of elastic tape during abduction and the baseline shoulder degree of subluxation, this intervention strategy also provided an improvement for this movement. Moreover, shoulder JPS plays an important role in feedback and feedforward controls during motor action to achieve specific roles for movement acuity, joint stability and coordination [4, 7, 9], which influence the upper limb performance. Thus, these improvement in shoulder JPS provided by elastic tape can improve joint stability and control of movement of UL. Furthermore, elastic tape would be an important strategy to facilitate the increase in sensory input, and should be associated with more repetitive task-specific training.

It is worth noting that the results of the present study are limited to the immediate effects of the elastic tape on shoulder JPS in subjects with chronic hemiparesis post-stroke with mild or moderate UL sensorimotor impairment. Thus, future studies that verify the effects of long-term elastic tape on joint position sense, as well as studies that verify short and long-term effects in other submodalities of proprioception, and/ or other phases of stroke are needed. In addition, the present study did not evaluate the effect of elastic tape on UL functional movements. Therefore, future studies are needed to verify the relationship between improvement in JPS and performance in the UL movements, as well as the effect of the elastic tape on daily UL activities. Furthermore, although an adequate sample size and large effect size were observed in the present study, for the correlation analysis, a larger sample size is required to further generalize our findings.

## Conclusions

Elastic tape applied to the paretic shoulder (anterior, middle, and posterior deltoid) improved JPS during abduction and flexion in chronic hemiparetic subjects, regardless of the level of UL sensorimotor impairment. However, these effects of elastic tape were influenced by the subluxation grade for shoulder abduction movements.

## Supporting Information

S1 Fig Schematic representation of the experimental design. JPS: Joint Position Sense.
(TIF)

S2 Fig Elastic tape application. (TIF)

S1 File CONSORT Checklist. (DOC)

S2 File Study protocol. (DOC)

S3 File Individual data. Individual data for ET and ST.
(XLS)

S1 Table Pilot data for sample size calculation. (DOC)

## References

[1] Feigin VL, Forouzanfar MH, Krishnamurthi R, Mensah GA, Connor M, Bennett DA, et al Global and regional burden of stroke during 1990–2010: findings from the Global Burden of Disease Study 2010. Lancet (London, England). 2014;383(9913):245–54. Epub 2014/01/23. 10.1016/s0140-6736(13)61953-4 PMC4181600 24449944

[2] Murray CJ, Vos T, Lozano R, Naghavi M, Flaxman AD, Michaud C, et al Disability-adjusted life years (DALYs) for 291 diseases and injuries in 21 regions, 1990–2010: a systematic analysis for the Global Burden of Disease Study 2010. Lancet. 2012;380(9859):2197–223. Epub 2012/12/19. 10.1016/S0140-6736(12)61689-4 23245608

[3] Hunter S, Crome P. Hand function and stroke. Reviews in Clinical Gerontology. 2002;12(01):68–81.

[4] Roijezon U, Clark NC, Treleaven J. Proprioception in musculoskeletal rehabilitation. Part 1: Basic science and principles of assessment and clinical interventions. Manual therapy. 2015;20(3):368–77. Epub 2015/02/24. 10.1016/j.math.2015.01.008 25703454

[5] Myers JB, Lephart SM. The role of the sensorimotor system in the athletic shoulder. J Athl Train. 2000;35(3):351–63. Epub 2006/03/25. 16558648 PMC1323397

[6] Hillier S, Immink M, Thewlis D. Assessing Proprioception: A Systematic Review of Possibilities. Neurorehabilitation and neural repair. 2015;29(10):933–49. Epub 2015/02/26. 10.1177/1545968315573055 25712470

[7] Riemann BL, Lephart SM. The Sensorimotor System, Part II: The Role of Proprioception in Motor Control and Functional Joint Stability. Journal of athletic training. 2002;37(1):80–4. Epub 2006/03/25. 16558671 PMC164312

[8] Proske U, Gandevia SC. The proprioceptive senses: their roles in signaling body shape, body position and movement, and muscle force. Physiological reviews. 2012;92(4):1651–97. Epub 2012/10/18. 10.1152/physrev.00048.2011 23073629

[9] Riemann BL, Lephart SM. The sensorimotor system, part I: the physiologic basis of functional joint stability. Journal of athletic training. 2002;37(1):71–9. Epub 2006/03/25. 16558670 PMC164311

[10] Dukelow SP, Herter TM, Bagg SD, Scott SH. The independence of deficits in position sense and visually guided reaching following stroke. Journal of neuroengineering and rehabilitation. 2012;9:72 Epub 2012/10/06. 10.1186/1743-0003-9-72 23035968 PMC3543214

[11] Dukelow SP, Herter TM, Moore KD, Demers MJ, Glasgow JI, Bagg SD, et al Quantitative assessment of limb position sense following stroke. Neurorehabilitation and neural repair. 2010;24(2):178–87. Epub 2009/10/02. 10.1177/1545968309345267 19794134

[12] Niessen MH, Veeger DH, Meskers CG, Koppe PA, Konijnenbelt MH, Janssen TW. Relationship among shoulder proprioception, kinematics, and pain after stroke. Archives of physical medicine and rehabilitation. 2009;90(9):1557–64. 10.1016/j.apmr.2009.04.004 19735784

[13] Niessen MH, Janssen TW, Meskers CGM, Koppe PA, Konijnenbelt MH, Veeger DH. Kinematics of the contralateral and ipsilateral shoulder: a possible relationship with post-stroke shoulder pain. Journal of Rehabilitation Medicine. 2008;40(6):482–6. 10.2340/16501977-0201 18509565

[14] Santos GL, Salazar LFG, Lazarin AC, Russo TL. Joint position sense is bilaterally reduced for shoulder abduction and flexion in chronic hemiparetic individuals. Topics in stroke rehabilitation. 2015;22(4):271–80. 10.1179/1074935714Z.0000000014 26258452

[15] Meyer S, De Bruyn N, Lafosse C, Van Dijk M, Michielsen M, Thijs L, et al Somatosensory Impairments in the Upper Limb Poststroke: Distribution and Association With Motor Function and Visuospatial Neglect. [published online ahead of print December 29, 2015]. Neurorehabilitation and neural repair. 2015 http://nnr.sagepub.com/content/early/2015/12/29/1545968315624779.full.pdf. Accessed January 25, 2015. 10.1177/1545968315624779 26719352

[16] Meyer S, Karttunen AH, Thijs V, Feys H, Verheyden G. How do somatosensory deficits in the arm and hand relate to upper limb impairment, activity, and participation problems after stroke? A systematic review. Physical therapy. 2014;94(9):1220–31. Epub 2014/04/26. 24764072 10.2522/ptj.20130271

[17] Colombo R, Sterpi I, Mazzone A, Delconte C, Pisano F. Improving proprioceptive deficits after stroke through robot-assisted training of the upper limb: a pilot case report study. Neurocase. 2016;22(2):191–200. Epub 2015/11/14. 10.1080/13554794.2015.1109667 26565132

[18] Doyle SD, Bennett S, Dudgeon B. Upper limb post-stroke sensory impairments: the survivor's experience. Disabil Rehabil. 2014;36(12):993–1000. Epub 2013/08/27. 10.3109/09638288.2013.825649 23971679

[19] Clark NC, Roijezon U, Treleaven J. Proprioception in musculoskeletal rehabilitation. Part 2: Clinical assessment and intervention. Manual therapy. 2015;20(3):378–87. Epub 2015/03/20. 10.1016/j.math.2015.01.009 25787919

[20] Kandel ER, Schwartz JH, Jessell TM. Principles of neural science: McGraw-Hill New York; 2000.

[21] Kase K, Wallis J, Kase T. Clinical therapeutic applications of Kinesio Taping Method. 2003.

[22] Lin JJ, Hung CJ, Yang PL. The effects of scapular taping on electromyographic muscle activity and proprioception feedback in healthy shoulders. J Orthop Res. 2011;29(1):53–7. Epub 2010/07/08. 10.1002/jor.21146 20607815

[23] Burfeind SM, Chimera N. Randomized Control Trial Investigating the Effects of Kinesiology Tape on Shoulder Proprioception. Journal of sport rehabilitation. 2015;24(4):405–12. Epub 2015/07/17. 26181196 10.1123/jsr.2014-0233

[24] Callaghan MJ, McKie S, Richardson P, Oldham JA. Effects of patellar taping on brain activity during knee joint proprioception tests using functional magnetic resonance imaging. Physical therapy. 2012;92(6):821–30. Epub 2012/01/28. 22282771 10.2522/ptj.20110209 PMC3367140

[25] Grampurohit N, Pradhan S, Kartin D. Efficacy of adhesive taping as an adjunt to physical rehabilitation to influence outcomes post-stroke: a systematic review. Topics in stroke rehabilitation. 2015;22(1):72–82. Epub 2015/03/18. 10.1179/1074935714Z.0000000031 25776123

[26] Pandian JD, Kaur P, Arora R, Vishwambaran DK, Toor G, Mathangi S, et al Shoulder taping reduces injury and pain in stroke patients: randomized controlled trial. Neurology. 2013;80(6):528–32. Epub 2013/01/25. 10.1212/WNL.0b013e318281550e 23345636

[27] Kalichman L, Frenkel-Toledo S, Vered E, Sender I, Galinka T, Alperovitch-Najenson D, et al Effect of kinesio tape application on hemiplegic shoulder pain and motor ability: a pilot study. International journal of rehabilitation research Internationale Zeitschrift fur Rehabilitationsforschung Revue internationale de recherches de readaptation. 2016;39(3):272–6. Epub 2016/04/15. 10.1097/MRR.0000000000000167 27075946

[28] Huang YC, Leong CP, Wang L, Wang LY, Yang YC, Chuang CY, et al Effect of kinesiology taping on the hemiplegic shoulder pain and functional outcomes in subacute stroke patients: a randomized controlled pilot study. European journal of physical and rehabilitation medicine. 2016. Epub 2016/08/31. 27575012

[29] Pillastrini P, Rocchi G, Deserri D, Foschi P, Mardegan M, Naldi MT, et al Effectiveness of neuromuscular taping on painful hemiplegic shoulder: a randomised clinical trial. Disability and rehabilitation. 2016;38(16):1603–9. Epub 2015/12/19. 10.3109/09638288.2015.1107631 26678717

[30] Lee D-H, Kim W-J, Oh J-S, Chang M. Taping of the elbow extensor muscle in chronic stroke patients: comparison between before and after three-dimensional motion analysis. Journal of Physical Therapy Science. 2015;27(7):2101–3. 10.1589/jpts.27.2101 26310566 PMC4540826

[31] De Baets L, Jaspers E, Janssens L, Van Deun S. Characteristics of neuromuscular control of the scapula after stroke: a first exploration. Frontiers in human neuroscience. 2014;8:933 Epub 2014/12/06. 10.3389/fnhum.2014.00933 25477805 PMC4235078

[32] Folstein MF, Folstein SE, McHugh PR. "Mini-mental state". A practical method for grading the cognitive state of patients for the clinician. Journal of psychiatric research. 1975;12(3):189–98. Epub 1975/11/01. 1202204 10.1016/0022-3956(75)90026-6

[33] Maki T, Quagliato E, Cacho E, Paz L, Nascimento N, Inoue M, et al Reliability study on the application of the Fugl-Meyer scale in Brazil. Brazilian Journal of Physical Therapy. 2006;10(2):177–83.

[34] Paci M, Nannetti L, Rinaldi L. Glenohumeral subluxation in hemiplegia: An overview. Journal of rehabilitation research and development. 2005;42(4):557 16320150 10.1682/jrrd.2004.08.0112

[35] Youdas JW, Carey JR, Garrett TR. Reliability of measurements of cervical spine range of motion—comparison of three methods. Physical therapy. 1991;71(2):98–104; discussion 5–6. Epub 1991/02/01. 1989013 10.1093/ptj/71.2.98

[36] Weir JP. Quantifying test-retest reliability using the intraclass correlation coefficient and the SEM. Journal of strength and conditioning research / National Strength & Conditioning Association. 2005;19(1):231–40. Epub 2005/02/12. 10.1519/15184.1 15705040

[37] Oldfield RC. The assessment and analysis of handedness: the Edinburgh inventory. Neuropsychologia. 1971;9(1):97–113. Epub 1971/03/01. 5146491 10.1016/0028-3932(71)90067-4

[38] Niessen MH, Veeger DH, Koppe PA, Konijnenbelt MH, van Dieën J, Janssen TW. Proprioception of the shoulder after stroke. Archives of physical medicine and rehabilitation. 2008;89(2):333–8. 10.1016/j.apmr.2007.08.157 18226659

[39] Yalcin E, Akyuz M, Onder B, Kurtaran A, Buyukvural S. Position Sense of the Hemiparetic and Non-Hemiparetic Ankle after Stroke: Is the Non-Hemiparetic Ankle also Affected? European neurology. 2012;68(5):294–9. 10.1159/000342025 23051834

[40] Niessen MH, Veeger DH, Koppe PA, Konijnenbelt MH, van Dieen J, Janssen TW. Proprioception of the shoulder after stroke. Archives of physical medicine and rehabilitation. 2008;89(2):333–8. Epub 2008/01/30. 10.1016/j.apmr.2007.08.157 18226659

[41] Gomez-Soriano J, Abian-Vicen J, Aparicio-Garcia C, Ruiz-Lazaro P, Simon-Martinez C, Bravo-Esteban E, et al The effects of Kinesio taping on muscle tone in healthy subjects: a double-blind, placebo-controlled crossover trial. Manual therapy. 2014;19(2):131–6. Epub 2014/05/16. 24829961 10.1016/j.math.2013.09.002

[42] Jaraczewska E, Long C. Kinesio taping in stroke: improving functional use of the upper extremity in hemiplegia. Topics in stroke rehabilitation. 2006;13(3):31–42. Epub 2006/09/22. 10.1310/33KA-XYE3-QWJB-WGT6 16987790

[43] Donec V, Varžaitytė L, Kriščiūnas A. The effect of Kinesio Taping on maximal grip force and key pinch force. Polish Annals of Medicine. 2012;19(2):98–105. 10.1016/j.poamed.2012.08.004.

[44] Silva Parreira Pdo C, Menezes Costa Lda C, Takahashi R, Hespanhol LC Junior, Motta Silva T, da Luz MA Junior, et al Do convolutions in Kinesio Taping matter? Comparison of two Kinesio Taping approaches in patients with chronic non-specific low back pain: protocol of a randomised trial. Journal of physiotherapy. 2013;59(1):52; discussion Epub 2013/02/20. 10.1016/S1836-9553(13)70147-4 23419916

[45] Michener LA, Kardouni JR, Lopes Albers AD, Ely JM. Development of a sham comparator for thoracic spinal manipulative therapy for use with shoulder disorders. Manual therapy. 2013;18(1):60–4. Epub 2012/08/14. 10.1016/j.math.2012.07.003 22883130

[46] Michener LA, Kardouni JR, Sousa CO, Ely JM. Validation of a sham comparator for thoracic spinal manipulation in patients with shoulder pain. Manual therapy. 2015;20(1):171–5. Epub 2014/09/28. 10.1016/j.math.2014.08.008 25261090 PMC4286506

[47] Olejnik S, Algina J. Measures of Effect Size for Comparative Studies: Applications, Interpretations, and Limitations. Contemporary educational psychology. 2000;25(3):241–86. Epub 2000/06/30. 10.1006/ceps.2000.1040 10873373

[48] Levine TR, Hullett CR. Eta squared, partial eta squared, and misreporting of effect size in communication research. Human Communication Research. 2002;28(4):612–25.

[49] Cohen J. Statistical Power Analysis for the Behavioral Sciences. 2nd edn Hillsdale, New Jersey: L. Erlbaum; 1988.

[50] Faraone SV. Interpreting Estimates of Treatment Effects: Implications for Managed Care. Pharmacy and Therapeutics. 2008;33(12):700–11. 19750051 PMC2730804

[51] Munro BH. Statistical methods for health care research: Lippincott Williams & Wilkins; 2005.

[52] Faul F, Erdfelder E, Lang AG, Buchner A. G*Power 3: a flexible statistical power analysis program for the social, behavioral, and biomedical sciences. Behavior research methods. 2007;39(2):175–91. Epub 2007/08/19. 17695343 10.3758/bf03193146

[53] Wu W-T, Hong C-Z, Chou L-W. The Kinesio Taping Method for Myofascial Pain Control. Evidence-Based Complementary and Alternative Medicine. 2015;2015. 10.1155/2015/950519 PMC4491400 26185522

[54] Vanti C, Bertozzi L, Gardenghi I, Turoni F, Guccione AA, Pillastrini P. Effect of taping on spinal pain and disability: systematic review and meta-analysis of randomized trials. Physical therapy. 2015;95(4):493–506. Epub 2014/11/22. 25413622 10.2522/ptj.20130619

[55] Chang WD, Chen FC, Lee CL, Lin HY, Lai PT. Effects of Kinesio Taping versus McConnell Taping for Patellofemoral Pain Syndrome: A Systematic Review and Meta-Analysis. Evidence-based complementary and alternative medicine: eCAM. 2015;2015:471208. Epub 2015/07/18. 26185517 10.1155/2015/471208 PMC4491411

[56] Taylor RL, O'Brien L, Brown T. A scoping review of the use of elastic therapeutic tape for neck or upper extremity conditions. Journal of hand therapy: official journal of the American Society of Hand Therapists. 2014;27(3):235–45; quiz 46. Epub 2014/05/06. 24794424 10.1016/j.jht.2014.03.004

[57] Parreira Pdo C, Costa Lda C, Hespanhol LC Jr., Lopes AD, Costa LO. Current evidence does not support the use of Kinesio Taping in clinical practice: a systematic review. Journal of physiotherapy. 2014;60(1):31–9. Epub 2014/05/27. 10.1016/j.jphys.2013.12.008 24856938

[58] Halseth T, McChesney JW, Debeliso M, Vaughn R, Lien J. The effects of kinesio taping on proprioception at the ankle. Journal of sports science & medicine. 2004;3(1):1–7. Epub 2004/03/01. PMC3896108 24497814

[59] Aarseth LM, Suprak DN, Chalmers GR, Lyon L, Dahlquist DT. Kinesio Tape and Shoulder-Joint Position Sense. J Athl Train. 2015;50(8):785–91. Epub 2015/06/20. 10.4085/1062-6050-50.7.03 26090707 PMC4629933

[60] Zanca GG, Mattiello SM, Karduna AR. Kinesio taping of the deltoid does not reduce fatigue induced deficits in shoulder joint position sense. Clinical biomechanics (Bristol, Avon). 2015;30(9):903–7. Epub 2015/08/26. 10.1016/j.clinbiomech.2015.07.011 26305054

[61] Hosp S, Bottoni G, Heinrich D, Kofler P, Hasler M, Nachbauer W. A pilot study of the effect of Kinesiology tape on knee proprioception after physical activity in healthy women. Journal of science and medicine in sport / Sports Medicine Australia. 2015;18(6):709–13. Epub 2014/10/02. 10.1016/j.jsams.2014.09.004 25270548

[62] Callaghan MJ, Selfe J, Bagley PJ, Oldham JA. The Effects of Patellar Taping on Knee Joint Proprioception. Journal of athletic training. 2002;37(1):19–24. Epub 2003/08/26. 12937439 PMC164303

[63] Callaghan MJ, Selfe J, McHenry A, Oldham JA. Effects of patellar taping on knee joint proprioception in patients with patellofemoral pain syndrome. Manual therapy. 2008;13(3):192–9. Epub 2007/02/14. 10.1016/j.math.2006.11.004 17296323

[64] Phadke V, Camargo P, Ludewig P. Scapular and rotator cuff muscle activity during arm elevation: A review of normal function and alterations with shoulder impingement. Revista brasileira de fisioterapia (Sao Carlos (Sao Paulo, Brazil)). 2009;13(1):1–9. Epub 2009/02/01. 10.1590/S1413-35552009005000012 PMC2857390 20411160

[65] Huang SW, Liu SY, Tang HW, Wei TS, Wang WT, Yang CP. Relationship between severity of shoulder subluxation and soft-tissue injury in hemiplegic stroke patients. Journal of rehabilitation medicine. 2012;44(9):733–9. Epub 2012/08/03. 10.2340/16501977-1026 22854896

