# Supplementary figures and images for: Elastic Tape Improved Shoulder Joint Position Sense in Chronic Hemiparetic Subjects: A Randomized Sham-Controlled Crossover Study
Source: PLoS One. 2017 Jan 18;12(1):e0170368. doi: 10.1371/journal.pone.0170368 (PMC5242462; doi:10.1371/journal.pone.0170368)

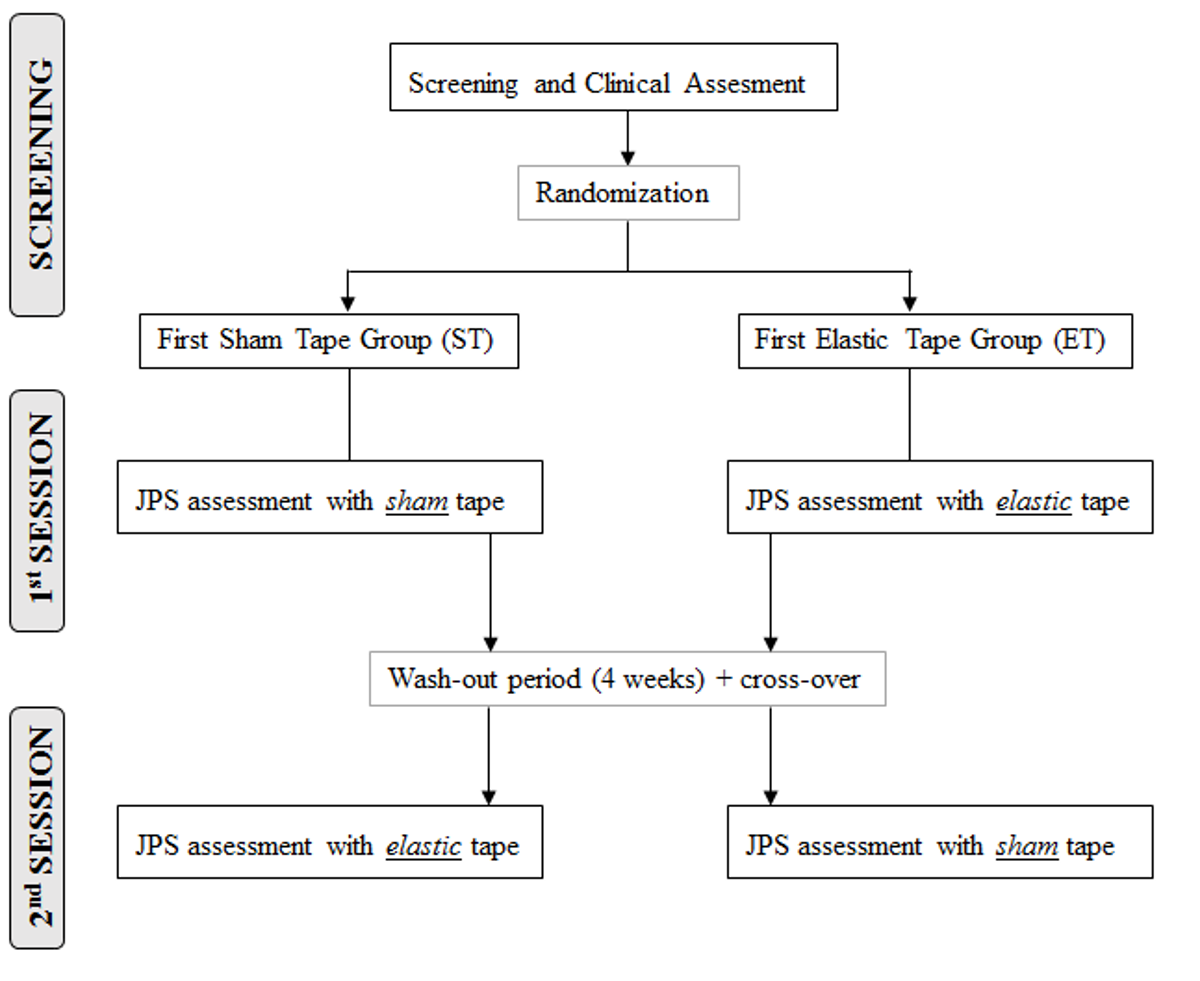

Supplement: S1 Fig — JPS: Joint Position Sense. (TIF) [file pone.0170368.s001.tif]

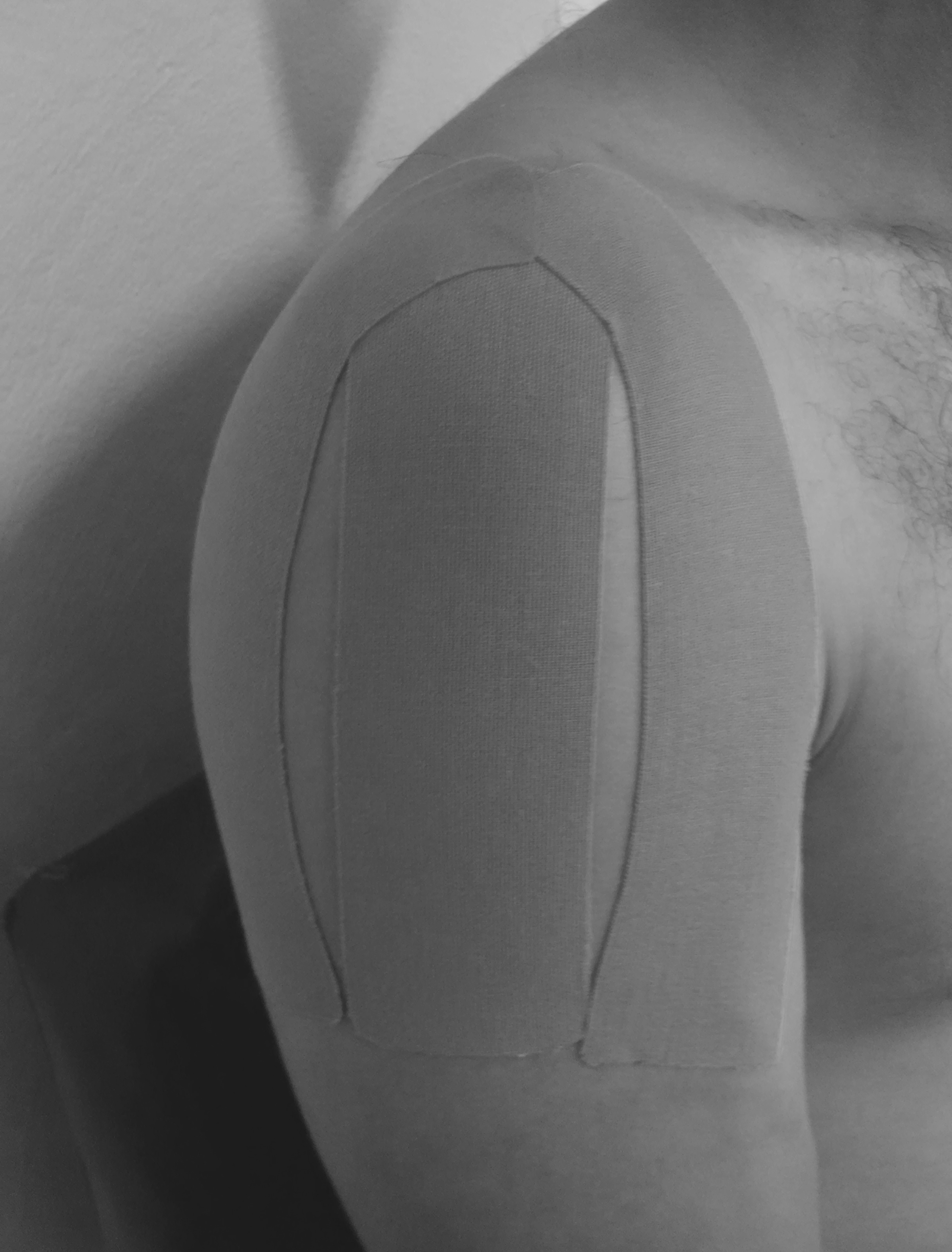

Supplement: S2 Fig — (TIF) [file pone.0170368.s002.tif]
