# Supplementary material for: Elastic Tape Improved Shoulder Joint Position Sense in Chronic Hemiparetic Subjects: A Randomized Sham-Controlled Crossover Study
Source: PLoS One. 2017 Jan 18;12(1):e0170368. doi: 10.1371/journal.pone.0170368 (PMC5242462; doi:10.1371/journal.pone.0170368)
Supplement: S1 Table — (DOC) [file pone.0170368.s006.doc]

**S1 Table. Pilot data for sample size calculation.**

|  | **Elastic tape intervention** | | **Sham tape intervention** | |
| --- | --- | --- | --- | --- |
| **Group** | **Pre** | **Post** | **Pre** | **Post** |
| **ET** | 9.92 (±3.63) | 2.75 (±2.23) | 10.92 (±5.44) | 10.67 (±4.15) |
| **ST** | 7.58 (±4.15) | 2.17 (±0.64) | 7.67 (±5.60) | 7.42 (±5.96) |

ET: elastic tape group. ST: Sham tape group. Data expressed as mean and standard deviation.
